# Erythropoietin Receptor Expression Is a Potential Prognostic Factor in Human Lung Adenocarcinoma

**DOI:** 10.1371/journal.pone.0077459

**Published:** 2013-10-14

**Authors:** Anita Rózsás, Judit Berta, Lívia Rojkó, László Z. Horváth, Magdolna Keszthelyi, István Kenessey, Viktória László, Walter Berger, Michael Grusch, Mir Alireza Hoda, Szilvia Török, Walter Klepetko, Ferenc Rényi-Vámos, Balázs Hegedűs, Balázs Döme, József Tóvári

**Affiliations:** 1 Department of Tumor Biology, National Koranyi Institute of Pulmonology, Budapest, Hungary; 2 Department of Bronchoscopy, National Koranyi Institute of Pulmonology, Budapest, Hungary; 3 Division of Thoracic Surgery, Medical University of Vienna, Vienna, Austria; 4 2^nd^ Department of Pathology, Semmelweis University, Budapest, Hungary; 5 Institute of Cancer Research, Department of Internal Medicine I, Medical University of Vienna, Vienna, Austria; 6 Department of Experimental Pharmacology, National Institute of Oncology, Budapest, Hungary; 7 Department of Thoracic Surgery, National Institute of Oncology, Budapest, Hungary; 8 Department of Measurement Technology and Industrial Electrical Engineering, Lund University, Lund, Sweden; Sapporo Medical University, Japan

## Abstract

Recombinant human erythropoietins (rHuEPOs) are used to treat cancer-related anemia. Recent preclinical studies and clinical trials, however, have raised concerns about the potential tumor-promoting effects of these drugs. Because the clinical significance of erythropoietin receptor (EPOR) signaling in human non-small cell lung cancer (NSCLC) also remains controversial, our aim was to study whether EPO treatment modifies tumor growth and if EPOR expression has an impact on the clinical behavior of this malignancy. A total of 43 patients with stage III–IV adenocarcinoma (ADC) and complete clinicopathological data were included. EPOR expression in human ADC samples and cell lines was measured by quantitative real-time polymerase chain reaction. Effects of exogenous rHuEPOα were studied on human lung ADC cell lines in vitro. In vivo growth of human ADC xenografts treated with rHuEPOα with or without chemotherapy was also assessed. In vivo tumor and endothelial cell (EC) proliferation was determined by 5-bromo-2’-deoxy-uridine (BrdU) incorporation and immunofluorescent labeling. Although EPOR mRNA was expressed in all of the three investigated ADC cell lines, rHuEPOα treatment (either alone or in combination with gemcitabine) did not alter ADC cell proliferation in vitro. However, rHuEPOα significantly decreased tumor cell proliferation and growth of human H1975 lung ADC xenografts. At the same time, rHuEPOα treatment of H1975 tumors resulted in accelerated tumor endothelial cell proliferation. Moreover, in patients with advanced stage lung ADC, high intratumoral EPOR mRNA levels were associated with significantly increased overall survival. This study reveals high EPOR level as a potential novel positive prognostic marker in human lung ADC.

## Introduction

Lung cancer, representing a major healthcare problem worldwide [1], is currently classified into two major groups: small cell and non-small cell lung cancer. The latter includes large-cell carcinoma, squamous-cell carcinoma and ADC. Approximately 85% of lung cancer patients have NSCLC and ADC accounts for more than 40% of all lung cancer cases [2]. Although targeted drugs have been incorporated into NSCLC therapeutic protocols, the overall prognosis of NSCLC patients remains poor: the five-year survival rate has been at a plateau of 15% for three decades. For this reason, a better understanding of the biological mechanisms involved in lung cancer development is urgently needed [3]. 

Cancer hypoxia has emerged as one of the key issues in lung cancer progression, as it has profound effects on angiogenesis, therapy resistance and cancer cell metabolism [4]. At the same time, cancer-related anemia occurs frequently in patients with lung cancer and leads to systemic and intratumoral hypoxia [5]. It is now established that hypoxia enhances the aggressiveness of cancer cells and promotes malignant progression. Moreover, tumor hypoxia has fundamental effects not only on the prognosis but on therapeutic responses as well [1]. Thus, attempts to correct the intratumoral oxygen status of lung cancer patients are justified. However, although rHuEPOs are effective drugs for correcting anemia, recent clinical trials have suggested inferior overall survival and/or locoregional control of tumors in patients receiving rHuEPO [6]. In addition, human and experimental studies have shown the co-expression of EPO/EPO receptor in various human malignancies and also demonstrated that the EPO/EPOR signaling plays a significant role in cancer cell proliferation, migration, invasiveness, and in the inhibition of apoptosis [7]. The EPOR expression in ECs has suggested that EPO may stimulate angiogenesis as well. 

As suggested in other recent studies, however, the overall direct effect of EPO/EPOR signaling on tumor progression and therapy is not straightforward. For instance, in a preclinical myeloma model, rHuEPO induced tumor regression and antitumor immune responses [8]. In another study, kidney carcinoma and myelomonocytic leukemia cell lines treated with rHuEPO exhibited an increase in apoptosis in response to chemotherapy [9]. 

To make the picture more complex, although certain clinical studies using commercially available anti-EPOR antibodies have suggested a relationship between EPOR expression and adverse clinical outcome following treatment with EPOs, most studies of EPOR detection in tumor tissues have provided false positive results because of the lack of EPOR specific antibodies (reviewed in ref [10].). Saintigny et al., for example, showed that EPO/EPOR co-expression is associated with poor survival in stage I NSCLC and suggested a potential role of endogenous EPO in the progression of these tumors [11]. Data from several other groups suggest, however, that the antibody used in the study of Saintigny et al. (C-20, SC-695; Santa Cruz, CA) is not valid for determining the EPOR status of tissue sections [12]. On the other hand, recent evidence also suggests that EPOR downregulation in NSCLC is compromised due to the lack of EPOR ubiquitination following EPO stimulation [13] and that increased pre-operative plasma EPO levels are associated with reduced survival in NSCLC patients [14]. EPO expression and EPO/EPOR co-expression were also found to be associated with poor loco-regional control and survival in irradiated stage II/III NSCLC patients [15]. Overall, because these findings warrant additional research of EPOR signaling in NSCLC, our aim was to examine the association between EPOR expression and the course of disease in human lung ADC. 

## Results

### EPOR expression and in vitro cell proliferation studies

**Figure 1:**
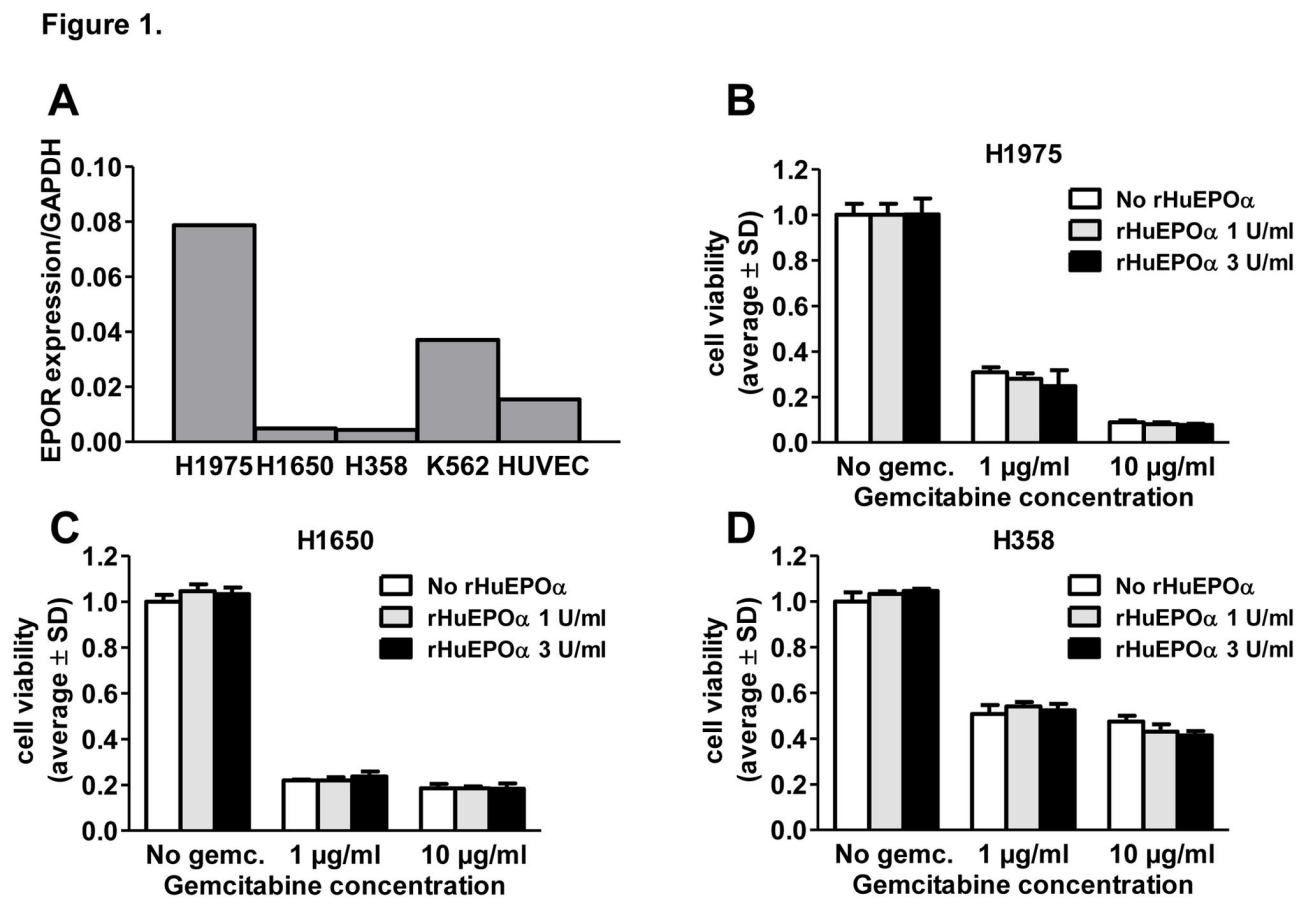
EPOR is expressed in human lung ADC cell lines but exogenous rHuEPOα does not modify ADC cell proliferation in vitro. (A) Real-time qRT-PCR demonstrating the expression of EPOR mRNA in human lung ADC cell lines and K562 and HUVEC cells as control. The highest EPOR expression level was detected in the H1975 ADC cell line. H1975 (B), H1650 (C) and H358 (D) cells were treated with rHuEPOα at different concentrations (1, 3 IU/ml) with or without gemcitabine (1, 10 µg/ml). Cell numbers were estimated at 48 hours by sulforhodamine B colorimetric assay. Although gemcitabine significantly decreased the proliferation of ADC cells (*p* < 0.001), rHuEPO treatment (either alone or in combination with gemcitabine) did not modify ADC cell proliferation in vitro.

EPOR mRNA levels of H1975, H1650 and H358 human ADCs were determined by using quantitative real-time PCR (qRT-PCR) analyses. HUVEC (human umbilical vein endothelial cell) and K562 erythroleukemia cell lines served as positive controls. H1650 and H358 lines expressed EPOR at low level. However, H1975 cells expressed EPOR mRNA at higher level than K562 cells (Figure 1A). 

To investigate whether EPO influences ADC cell growth in an autocrine manner, the effect of treatments with different rHuEPOα doses was studied on three human ADC cell lines. Importantly, rHuEPOα did not stimulate the in vitro proliferation rate of any of the three cell lines when compared with untreated cells after 48 hours (Figure 1 B-D).

As expected, treatment with gemcitabine (1, 10 µg/ml) significantly decreased cell proliferation in all examined human ADC lines (*p* < 0.001). rHuEPOα alone did not alter cell proliferation and the anti-proliferative effect of gemcitabine was not affected by rHuEPOα at any concentrations (Figure 1 B-D). 

### In vivo effect of rHuEPOα and gemcitabine treatments

**Figure 2:**
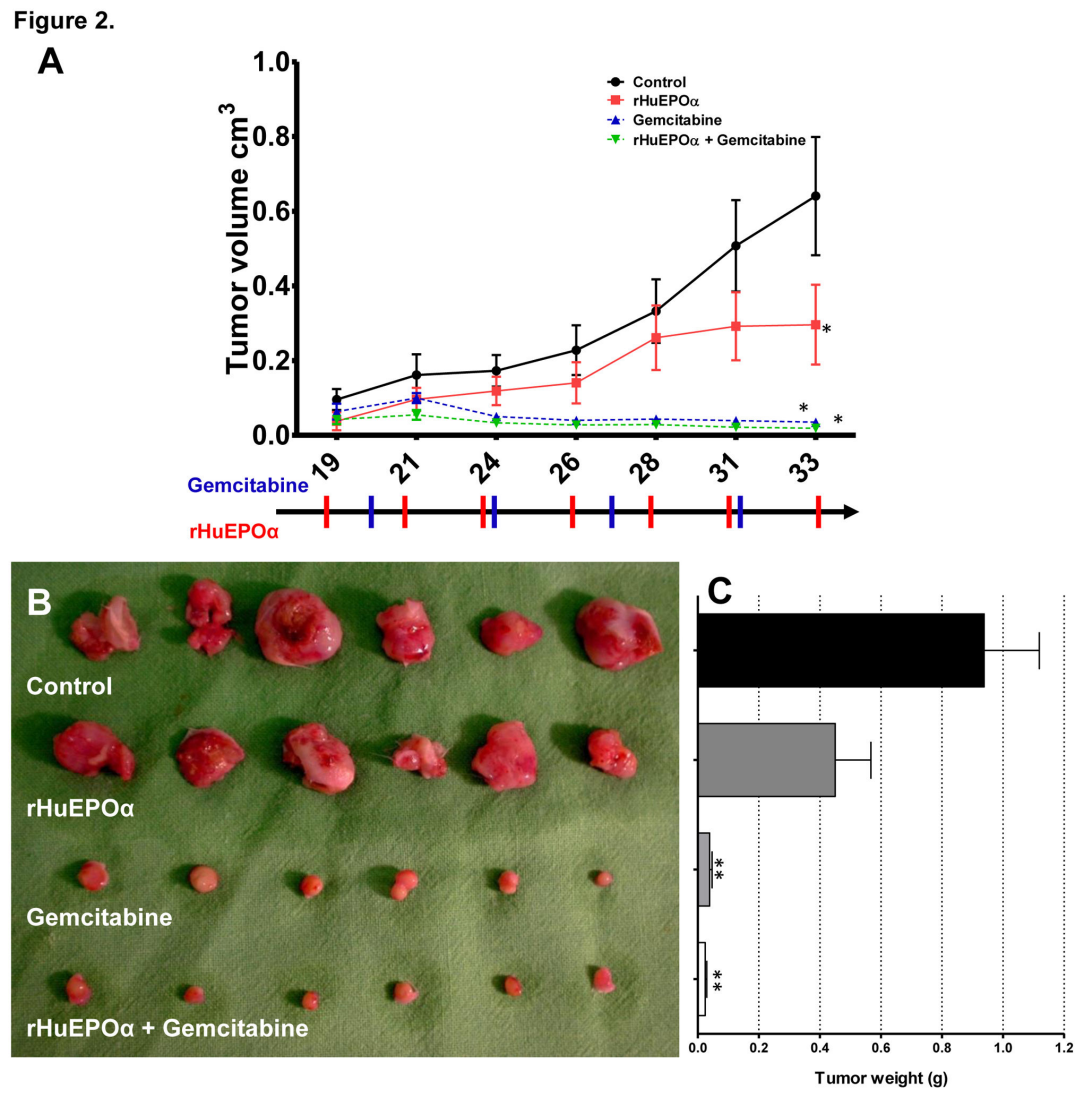
Exogenous rHuEPOα reduced in vivo growth of human lung adenocarcinoma cells in SCID mice. (A) Growth curves and (C) tumor weights of control, rHuEPOα (150 IU/kg), gemcitabine (100 mg/kg) and rHuEPOα (150 IU/kg) plus gemcitabine (100 mg/kg) groups; *p < 0.05 versus control; **p < 0.001, versus control. (B) Surgically removed H1975 xenografts at the end of the experiment (day 33).

Next, we sought to study the effect of rHuEPOα on the in vivo growth of the H1975 cell line that showed the highest in vitro EPOR expression. Therefore, in our next set of experiments, the growth rates of subcutaneously implanted H1975 tumors after rHuEPOα or gemcitabine treatments alone or in combination were assessed. Tumor growth was significantly decreased in mice treated with gemcitabine alone. Surprisingly, a less robust but still significant growth-inhibitory effect of rHuEPOα was observed when administered alone. However, no additional synergistic effects could be achieved when the two drugs were given in combination (Figure 2 A-B). The mean tumor weights in the control, rHuEPO, gemcitabine and gemcitabine plus rHuEPO treated groups were 0.938 g, 0.45 g, 0.039 g and 0.024 g, respectively (Figure 2 C). 

**Figure 3:**
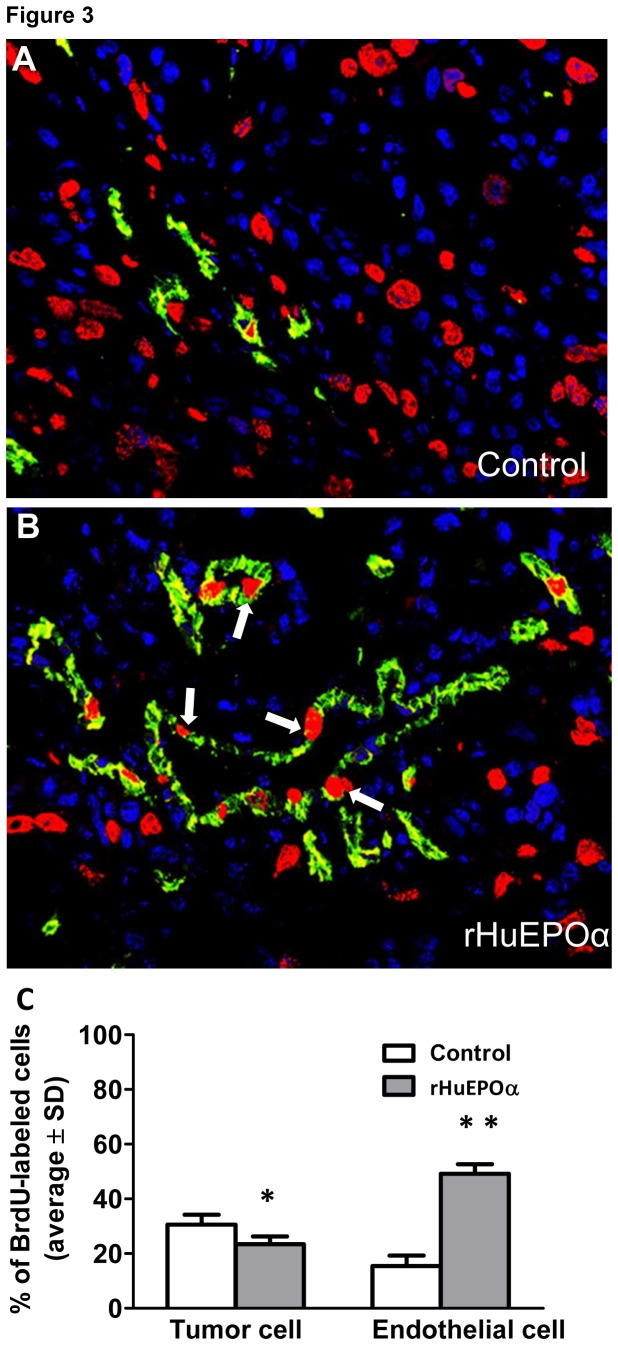
Effect of rHuEPOα and gemcitabine treatments on the proliferation of endothelial and tumor cells in H1975 xenograft tumors. Representative immunofluorescent images of tumors from control (A) and rHuEPOα-treated (B) mice. Tumor sections are stained for the endothelial marker, CD31 (green), the proliferation-associated marker, BrdU (red) and for TOTO-3 (blue) highlighting EC as well as tumor cell nuclei. Arrows in (B) point at proliferating endothelial cells. (C) Labeling index of tumor and endothelial cells in 33-day-old rHuEPOα-treated or control H1975 tumors. *p = 0.021, versus controls; **p < 0.001, versus controls.

In mice treated with rHuEPOα alone, the proliferation index of H1975 cells and mouse blood vessel ECs was determined by BrdU labeling (Figure 3 A-B). rHuEPOα not only resulted in accelerated EC proliferation in vivo (Figure 3 C) but surprisingly it also significantly decreased the in vivo growth rate of the high EPOR receptor-expressing H1975 NSCLC cells (Figure 2 A-B). 

### Association of bronchoscopy brush EPOR mRNA expressions with clinicopathological parameters

To determine the clinical relevance of tumor tissue EPOR expression, we performed comparative statistical analysis of bronchial brush EPOR mRNA expression and clinicopathological variables. In line with the findings of Yasuda et al. [16], our preliminary studies revealed a significant variability in normal lung tissue EPOR expressions. Therefore, we normalized tumor tissue EPOR expressions to the patient-matched normal endobronchial EPOR expression levels (T/N) (Figure S1). No significant associations with age, smoking history, gender, stage or treatment were detected (Table 1).

**Table 1:** Correlation of clinicopathologic features and EPOR expression in ADC (n=43).

	**No. of patients with ADC (%)**	**EPOR expression (T/N)**		***P value***
		**Low** (**%**)^a^	**High** (**%**)^a^	
**All patients**	**43** (100%)	**28** (65.1%)	**15** (34.9%)	
**Age** (**years**) ^b^				
61<	20 (46.5)	13 (46.4)	7 (46.7)	
61≥	23 (53.5)	15 (53.6)	8 (53.3)	**^0.99 c^**
Smoking*				
Non-smoker	2 (4.7)	1 (3.6)	1 (6.7)	
Current or ex-smoker	30 (69.8)	21 (75)	9 (60)	**^0.53 d^**
**Gender**				
Male	19 (44.2)	13 (46.4)	6 (40)	
Female	24 (55.8)	15 (53.6)	9 (60)	**^0.69 c^**
**Stage**				
III	14 (32.6)	9 (32.1)	5 (33.3)	
IV	29 (67.4)	17 (60.7)	10 (66.7)	**^0.93 c^**
Treatment**				
PBC	15 (34.9)	7 (25)	8 (53.3)	
RCT	12 (27.9)	10 (35.7)	2 (13.3)	
PT	9 (20.9)	6 (21.4)	3 (20)	
Surgery	3 (7)	2 (7.1)	1 (6.7)	
S+CT and/or RT	1 (2.3)	1 (3.6)	0 (0)	**^0.34 c^**

^a^ T/N is the ratio of tumor tissue and normal tissue expression. Low expression is defined below one; ^b^ Cut-off value is median value; ^c^ Chi-square test; ^d^ Fisher’s exact test; Data shown in parentheses are column percentages; ADC, adenocarcinoma; PBC, Platinum-Based chemotherapy; RCT, Radiochemotherapy; PT, Palliative therapy; CT, chemotherapy; RT, radiotherapy; * 11 ADC patients had unknown smoking status; ** there was no available information in case of 3 ADC patients

### EPOR expression level as a prognostic marker

**Figure 4:**
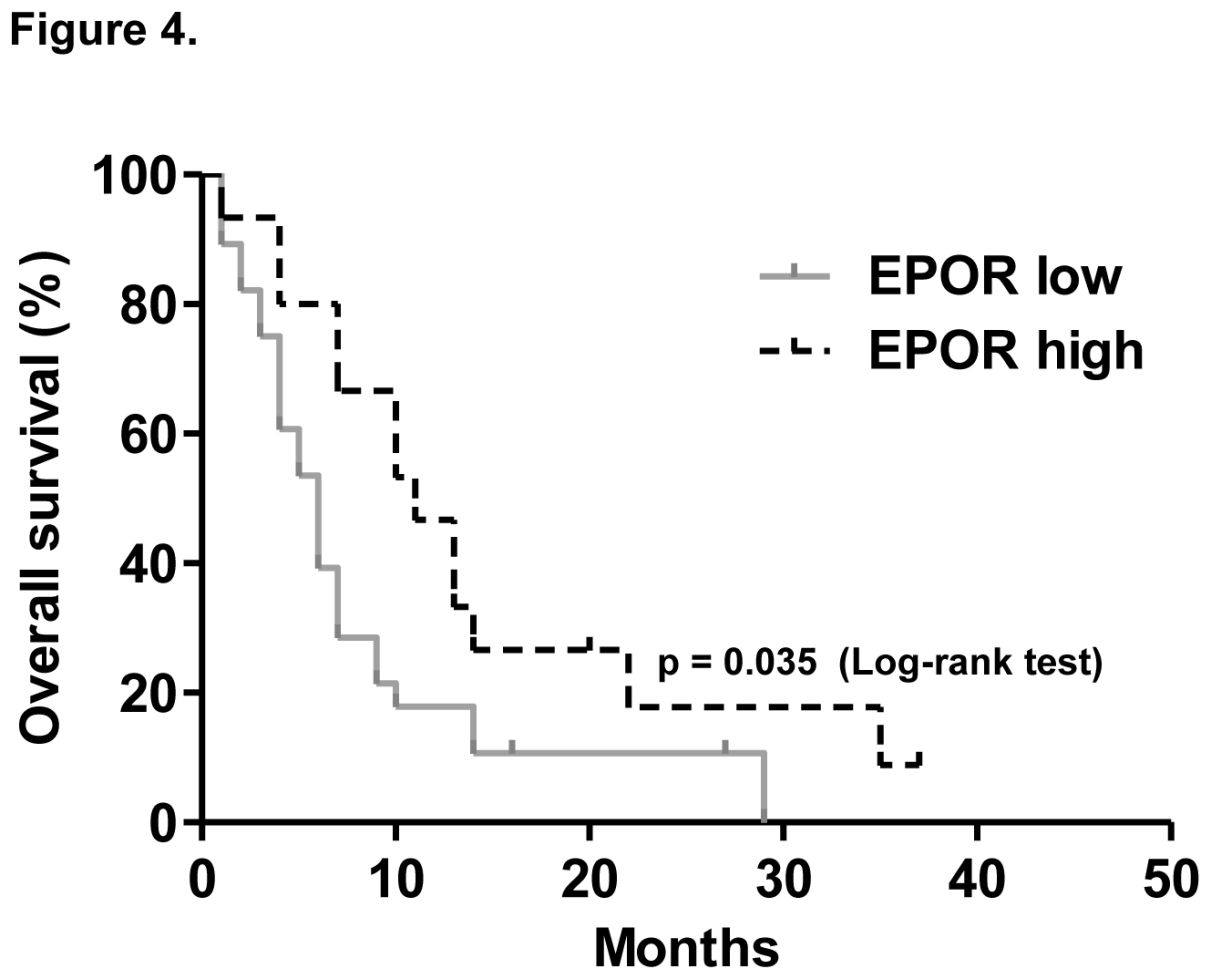
Low EPOR expression is associated with poor prognosis in advanced stage human pulmonary adenocarcinoma. (A) Kaplan-Meier curves for the overall survival of the entire patient population with stage III-IV ADC (n = 43), according to high and low EPOR expression as determined by the ratio of tumor and normal bronchoscopy brush EPOR mRNA expressions based on quantitative real-time PCR.

Next we used Kaplan-Meier analysis to calculate the overall survival rates for advanced stage ADC patients with low and high EPOR levels. We elucidated that ADC patients with high EPOR levels had significantly longer survival than those with low EPOR expression (median survival was 11 versus 6 months, respectively; *p* = 0.035; Figure 4). Multivariate analysis (including standard prognostic variables, such as age, gender, tumor stage and smoking history) also indicated that pretreatment EPOR levels predicted outcome independent of other variables (*p* = 0.031; Table 2). A further independent prognostic factor related to poor survival was patients' age and gender (*p* = 0.012 and 0.018, respectively, Table 2).

**Table 2:** Multivariate analysis of various prognostic factors in patients with advanced stage lung ADC.

**Prognostic factor**	**RR**	**95% CI**	***P***
**Age**	1.077	(1.017-1.142)	0.012
**Gender** (male vs. female)	0.364	(0.157-0.843)	0.018
**Stage** (III vs. IV)	0.615	(0.291-1.302)	0.204
**Smoking (ever smokers vs never-smokers)**	0.67	(0.258-1.741)	0.342
**EPOR (low vs. high)**	0.431	(0.201-0.926)	0.031

RR, relative risk; CI, confidence interval

## Discussion

Although EPOR is widely expressed in malignant tumors including human NSCLC, the significance of EPOR signaling in this malignancy is not fully elucidated. Therefore, we assessed EPOR mRNA expression levels in bronchoscopy brush samples of stage III-IV ADC patients, and investigated whether these levels might be related to patient’s clinicopathological variables and/or prognosis. This study presents the novel finding that advanced stage pulmonary ADC patients with high EPOR mRNA expressions have significantly better prognosis than those with low EPOR levels. 

EPOR is expressed in non-hematopoietic tissues suggesting that EPO has pleiotropic effects extending well beyond erythropoiesis. EPOR is also expressed in various cancer cell lines (including NSCLC) and in human tumor tissues including stromal components such as the vasculature [7]. Iatrogenic effects of rHuEPO via EPOR signaling resulting in potentially accelerated tumor cell proliferation and angiogenesis may, thus, compete with rHuEPO’s beneficial effects. In accordance with results of previous studies [13,17–20], although EPOR was also present at the mRNA level in all our investigated human lung ADC cell lines, exogenous rHuEPOα (either alone or in combination with gemcitabine) did not have an effect on ADC cell growth in vitro. Unexpectedly, however, rHuEPOα alone significantly decreased tumor cell proliferation and growth of human xenograft tumors formed by H1975 cells (with the highest EPOR expression among the investigated ADC cell lines). The reasons for this in vivo antitumoral effect of rHuEPOα are not fully understood. One possible explanation can be that rHuEPOα corrected intratumoral hypoxia which, in turn, might have inhibited the activation of the major hypoxia regulator, hypoxia-inducible factor (HIF)-1α [21]. The observed parallel and significant increase of tumor EC proliferation (and supposedly the consequent increase in intratumoral capillary surface) in H1975 xenografts supports this scenario. In line with this, our group has demonstrated previously that in human colon (HT25) and epidermoid carcinoma (A431) xenograft models, rHuEPOα administration significantly decreased tumoral hypoxia and HIF-1α and vascular endothelial growth factor (VEGF) expressions but had no effect on tumor growth. At the same time, rHuEPOα significantly increased the proliferation of tumor-associated ECs and the diameter of intratumoral blood vessels. The increased vessel surface resulted in improved drug delivery to tumor cells and augmented the antitumoral effects of chemotherapy [22]. In a more recent study, we also found that rHuEPOα did not modify the in vitro proliferation of EPOR expressing A431 tumor cells but enhanced the effect of irradiation on proliferation, apoptosis and clonogenic capacity. In the same study, treatment with rHuEPOα alone decreased tumoral HIF-1α expression but had no effect on tumor growth. However, rHuEPOα significantly increased the efficacy of radiotherapy in vivo [23]. 

Association between EPOR signaling and disease outcome, including survival, has hitherto been studied in only a small number of NSCLC patients [11,24]. In addition, the only two studies on the prognostic value of EPOR expression in human NSCLC samples [24] were based on tumor immunohistochemistry data generated with anti-EPOR antibodies the specificity of which has been questioned so far [12]. By using the most frequently used antibodies (M-20, Santa Cruz Biotechnologies; MAB307, R&D Systems; and A82, kindly provided by Amgen), we also attempted to measure EPOR expression at the protein level in our human lung ADC cell lines. However, we either detected various nonspecific signals (M-20 and MAB307) or failed to show any signs of EPOR protein expression (A82) by Western blot analysis in our ADC cell lines (data not shown). This observation is in accordance with the data of Elliott et al. who found that the currently available anti-EPOR antibodies have limited utility for detecting EPOR [25]. It is also important to mention that the posttranslational processing and alternative splicing also modify the structure of EPOR [26,27]. This might be an additional explanation for the lack of specific anti-EPOR antibodies. Our analysis of EPOR expression in human ADC samples based on qRT-PCR, thus, provides a more reliable picture on the role of EPOR signaling in human ADC. More precisely, in addition to the demonstration of significant in vivo growth-inhibitory effect of rHuEPOα alone, this prospective study also presents the novel observation that qRT-PCR measurement of EPOR expression in bronchial brushes is a useful tool to predict outcomes in patients with advanced stage lung ADC. During the follow-up period, a significantly higher incidence of death from lung ADC was found in patients with low pretreatment EPOR levels as compared with those of high EPOR levels. Although large-scale meta-analyses of clinical trials on erythropoiesis-stimulating agents in various tumor types [28], including NSCLC [29], suggest no effect of these drugs on patients' prognosis, our results, therefore, support an effect of rHuEPOα either directly or indirectly reducing pulmonary ADC progression. The current preliminary study, however, has to be confirmed in further studies in additional cohorts of patients with lung ADC. 

## Materials and Methods

### Cell lines

The H358, H1975 and H1650 ADC lines and the K562 erythroleukemia cells were obtained from the American Type Culture Collection (Manassas, VA). All cell lines were cultured in RPMI-1640 (Sigma Chemical Co., St. Louis, MO) supplemented with 10% fetal bovine serum (Sigma) and 100 U/ml penicillin-100 Ag/ml streptomycin (Sigma). HUVECs were isolated from fresh-term umbilical cords as described previously [30], and were cultured in EBM-2 Basal Medium (Lonza Cologne GmbH, Walkersville, MD) supplemented with EC growth media (Lonza). All cell lines were maintained in at 37°C in a humidified incubator with 5% CO2. 

### Human samples and clinicopathological data

All patients gave their written informed consent and the human sample collection was approved by the Scientific and Research Ethics Committee of the Medical Research Council of Hungary (153/PI/10; 2521-0/2010-1018EKU). A total of 43 patients with stage III-IV ADC treated in the National Koranyi Institute of TB and Pulmonology, Hungary from January 2009 to December 2010 were included into the study.

For qRT-PCR analyses, bronchoscopy brushes of patients with ADC were obtained and stored at -80°C until total RNA isolation. In each patient two brushings were done, one from normal endobronchial surface distant from the tumor site, and an other from the tumor tissue. Both samples underwent examination by an expert cytologist as well. 

There were 19 male and 24 female patients with a median age of 61 (Table 1). The cases were staged according to operative and pathologic findings based on The American Joint Committee on Cancer/Union Internationale Contre le Cancer TNM classification. 

### Measurement of EPOR expression on human samples and tumor cell lines

The total mRNA of the samples and the cell lines were extracted by TRI Reagent (Sigma). The extracted RNA was purified with DNA-free DNase kit (Ambion, Austin, TX). 12,5 μl of total RNA were reverse transcribed from each samples using deoxy-NTPs, a mixture of random primer and oligodT, RNasin ribonuclease inhibitor (20 U/reaction; Promega, Madison, WI), reverse transcriptase buffer and M-MLV reverse transcriptase (200 U/reaction, Sigma). They were incubated for 50 min at 37°C and then at 85°C for 10 min. The reverse transcription and the lack of genomic DNA contamination was demonstrated by primers specific for β-actin transcripts (sense: 5’-GTG GGG CGC CCC AGG CAC CCA-3’ and antisense: 5’-CTC CTT AAT GTC ACG CAC GAT TTC-3’). The real-time PCR reaction was run on the Applied Biosystems 7500 PCR machine using TaqMan Universal PCR Master mix and TaqMan premade gene expression assays; EPOR: Hs000181092_m1; β-actin: Hs03023880_g1; GAPDH: Hs02786624_g1 (Applied Biosystems, Foster City, CA). The cycling parameters were as follows: 2 minutes at 50°C, 40 cycles of 10 seconds at 95 °C, 1 minute at 60°C and 1 minute at 72°C. EPOR expression levels of human samples were determined after normalization to β-actin expression and to GAPDH in case of cell lines. The tumor tissue EPOR expressions were normalized to the patient-matched normal endobronchial EPOR expression levels (T/N).

### In vitro cell proliferation assay

H358, H1975 and H1650 cells (5x10^3^ per well) were plated into 96-well tissue culture plate (6-6 parallel from each cell line/treatment) in RPMI-1640 medium containing 10% fetal bovine serum. After 24 hours, cell were treated for 48 hours in 150μl medium containing 3% FBS. Treatments were as follows: rHuEPOα (Jannsen-Cilag, Shaffhausen, Switzerland) at 1 and 3 IU/ml, gemcitabine (Gemzar, Lilly, France) at 1, 10 µg/ml and the combination of rHuEPOα (1, 3 IU/ml) and gemcitabine (1, 10 µg/ml) simultaneously. After 48 hours, cell growth was assessed by sulforhodamine B (SRB) colorimetric assay. Briefly, the culture medium was aspirated prior to precipitation of proteins with 10% trichloroacetic acid for 1 hour at 4°C. Then cells were washed five times with deionized water and stained for 15 minutes with 0.4% SRB dye in 1% acetic acid. To remove unbound stain, plates were washed with 1% acetic acid and left to dry at room temperature. Finally, the stained proteins were solubilized in 10 mM Tris buffer. Absorbance was measured at 570 nm by Varioskan microplate reader (Thermo Fisher Scientific Inc., Waltham, MA, USA). The experiments were repeated independently three times.

### Xenograft tumors

The backs of female SCID (CB17/ICR-*Prkdc* ^*scid*^) mice were subcutaneously inoculated with 1,5x10^6^ H1975 tumor cells. All animal experiments were conducted following standards and procedures approved by the Animal Care and Use Committee of the National Institute of Oncology, Budapest (22.1/1268/3/2010).

### In vivo rHuEPOα and gemcitabine treatment

The first group of SCID mice was treated intraperitoneally with rHuEPOα at human-equivalent dose (150 IU/kg in physiological salt solution, final volume 0.1 ml) three times per week from day 5 till day 33. The second group was treated intraperitoneally four times with gemcitabine (100 mg/kg) [31–33] on the day 20, 24, 27 and 31. The third group received rHuEPOα intraperitoneally at human-equivalent dose thrice per week from day 5 till day 33 and gemcitabine (100 mg/kg) on day 20, 24, 27 and 31. Control animals were given physiological salt solution only. Tumor volumes were measured thrice per week from day 19 with a caliper and expressed in cm^3^ by the formula for the volume of a prolate ellipsoid [length × width^2^ × π/6]. 

### Measurement of proliferation index

rHuEPOα-treated, tumor-bearing animals were injected intraperitoneally with BrdU (200 mg/kg, Sigma). After 1 hour incubation, tumors were removed and snap frozen. 5 μm frozen sections were cut and immunostained, as described previously [34]. BrdU-positive cells were detected by anti-BrdU monoclonal antibody (Becton Dickinson, Bioscience, San Jose, CA) and TRITC-conjugated anti-mouse IgG (1:100, Sigma). Slides were then incubated with monoclonal rat anti-mouse CD31 antibody (1:20, Becton Dickinson) followed by biotinylated anti-rat IgG and streptavidin-FITC (Vector Laboratories, Burlingame, CA) to discriminate ECs from tumor cells. The nuclei were stained with TOTO-3 iodide (Invitrogen, Carlsbad, CA, USA). The BrdU labeling index was determined by counting non-labeled and labeled H1975 and mouse EC nuclei in independent intratumoral areas. The labeling index was calculated by dividing the number of labeled nuclei by the total number of nuclei counted, as described previously [22,34–37].

### Statistical analysis

To determine statistical differences between two groups t-test was used. ANOVA was used with the *post hoc* Scheffé- or Bonferroni-tests for the comparison of more than two groups. For the animal experiments we used the non-parametric Mann-Whitney U-test. Categorical data were compared using Fishers’ exact probability and chi-square tests. Overall survival analyses were done using the Kaplan-Meier method. Overall survival intervals were determined as the time period from initial diagnosis to the time of death. The comparison between survival functions for different strata was assessed with the log-rank statistics. Multivariate analysis of the clinical parameters was performed using Cox’s regression model. Statistical significance was determined when *P* values were <0.05. Statistical analysis was performed using GraphPad Prism 5.0 software (GraphPad Inc., San Diego, CA, USA) for preclinical data and Statistica 9.0 software for the clinical data (StatSoft, Tulsa, OK, USA).

## Supporting Information

Figure S1 **EPOR expression measured in tumoral (**T**) and normal (**N**) pairs of tissue samples of pulmonary ADC patients.** For qRT-PCR measurement, RNA was isolated from bronchoscopy brushes of ADC patients. Two samples were taken in each patient, one from the tumor site (T), and an other from the tumor-free endobronchial surface (N).
(TIF)

## References

[1] Jemal A, Bray F, Center MM, Ferlay J, Ward E et al. (2011) Global cancer statistics. CA Cancer J Clin 61: 69-90. doi:10.3322/caac.20107. PubMed: 21296855. 21296855

[2] Kadara H, Kabbout M, Wistuba II (2012) Pulmonary adenocarcinoma: a renewed entity in 2011. Respirology 17: 50-65. doi:10.1111/j.1440-1843.2011.02095.x. PubMed: 22040022. 22040022 PMC3911779

[3] Kelly K, Huang C (2008) Biological agents in non-small cell lung cancer: a review of recent advances and clinical results with a focus on epidermal growth factor receptor and vascular endothelial growth factor. J Thorac Oncol 3: 664-673. doi:10.1097/JTO.0b013e3181758141. PubMed: 18520811. 18520811

[4] Bertout JA, Patel SA, Simon MC (2008) The impact of O2 availability on human cancer. Nat Rev Cancer 8: 967-975. doi:10.1038/nrc2540. PubMed: 18987634. 18987634 PMC3140692

[5] Crawford J, Kosmidis PA, Hirsch FR, Langer CJ (2006) Targeting anemia in patients with lung cancer. J Thorac Oncol 1: 716-725. doi:10.1097/01243894-200609000-00020. PubMed: 17409943. 17409943

[6] Bohlius J, Schmidlin K, Brillant C, Schwarzer G, Trelle S et al. (2009) Recombinant human erythropoiesis-stimulating agents and mortality in patients with cancer: a meta-analysis of randomised trials. Lancet 373: 1532-1542. doi:10.1016/S0140-6736(09)60502-X. PubMed: 19410717. 19410717

[7] Tóvári J, Pirker R, Tímár J, Ostoros G, Kovács G et al. (2008) Erythropoietin in cancer: an update. Curr Mol Med 8: 481-491. doi:10.2174/156652408785747979. PubMed: 18781955. 18781955

[8] Mittelman M, Neumann D, Peled A, Kanter P, Haran-Ghera N (2001) Erythropoietin induces tumor regression and antitumor immune responses in murine myeloma models. Proc Natl Acad Sci U S A 98: 5181-5186. doi:10.1073/pnas.081275298. PubMed: 11309490. 11309490 PMC33184

[9] Carvalho G, Lefaucheur C, Cherbonnier C, Métivier D, Chapel A et al. (2005) Chemosensitization by erythropoietin through inhibition of the NF-kappaB rescue pathway. Oncogene 24: 737-745. doi:10.1038/sj.onc.1208205. PubMed: 15580299. 15580299

[10] Fandrey J (2008) Erythropoietin receptors on tumor cells: what do they mean? Oncologist 13 Suppl 3: 16-20. doi:10.1634/theoncologist.13-S3-16. PubMed: 18458120. 18458120

[11] Saintigny P, Besse B, Callard P, Vergnaud AC, Czernichow S et al. (2007) Erythropoietin and erythropoietin receptor coexpression is associated with poor survival in stage I non-small cell lung cancer. Clin Cancer Res 13: 4825-4831. doi:10.1158/1078-0432.CCR-06-3061. PubMed: 17699861. 17699861

[12] Brown WM, Maxwell P, Graham AN, Yakkundi A, Dunlop EA et al. (2007) Erythropoietin receptor expression in non-small cell lung carcinoma: a question of antibody specificity. Stem Cells 25: 718-722. PubMed: 17110616. 17110616 10.1634/stemcells.2006-0687

[13] Dunlop EA, Percy MJ, Boland MP, Maxwell AP, Lappin TR (2006) Induction of signalling in non-erythroid cells by pharmacological levels of erythropoietin. Neurodegener Dis 3: 94-100. doi:10.1159/000092099. PubMed: 16909043. 16909043

[14] Paul I, Lappin TR, Maxwell P, Graham AN (2006) Pre-operative plasma erythropoietin concentration and survival following surgery for non-small cell lung cancer. Lung Cancer 51: 329-334. doi:10.1016/j.lungcan.2005.10.020. PubMed: 16412529. 16412529

[15] Rades D, Setter C, Dahl O, Schild SE, Noack F (2011) Prognostic impact of erythropoietin expression and erythropoietin receptor expression on locoregional control and survival of patients irradiated for stage II/III non-small-cell lung cancer. Int J Radiat Oncol Biol Phys 80: 499-505. doi:10.1016/j.ijrobp.2010.02.003. PubMed: 20646855. 20646855

[16] Yasuda Y, Hara S, Hirohata T, Koike E, Yamasaki H et al. (2010) Erythropoietin-responsive sites in normal and malignant human lung tissues. Anat Sci Int 85: 204-213. doi:10.1007/s12565-010-0081-7. PubMed: 20397063. 20397063

[17] Berdel WE, Danhauser-Riedl S, Oberberg D, Zafferani M (1992) Effects of hematopoietic growth factors on malignant nonhematopoietic cells. Semin Oncol 19: 41-45. PubMed: 1553574. 1553574

[18] Mundt D, Berger MR, Bode G (1992) Effect of recombinant human erythropoietin on the growth of human tumor cell lines in vitro. Micro-titertec-tetrazolium assay. Arzneimittelforschung 42: 92-95. PubMed: 1586390. 1586390

[19] Rosti V, Pedrazzoli P, Ponchio L, Zibera C, Novella A, et al. (1993) Effect of recombinant human erythropoietin on hematopoietic and non-hematopoietic malignant cell growth in vitro. Haematologica 78: 208-212 8294051

[20] Berdel WE, Oberberg D, Reufi B, Thiel E (1991) Studies on the role of recombinant human erythropoietin in the growth regulation of human nonhematopoietic tumor cells in vitro. Ann Hematol 63: 5-8. doi:10.1007/BF01714953. PubMed: 1878424. 1878424

[21] Belozerov VE, Van Meir EG (2005) Hypoxia inducible factor-1: a novel target for cancer therapy. Anti Cancer Drugs 16: 901-909. doi:10.1097/01.cad.0000180116.85912.69. PubMed: 16162966. 16162966

[22] Tóvári J, Gilly R, Rásó E, Paku S, Bereczky B et al. (2005) Recombinant human erythropoietin alpha targets intratumoral blood vessels, improving chemotherapy in human xenograft models. Cancer Res 65: 7186-7193. doi:10.1158/0008-5472.CAN-04-2498. PubMed: 16103069. 16103069

[23] Lövey J, Bereczky B, Gilly R, Kenessey I, Rásó E et al. (2008) Recombinant human erythropoietin alpha improves the efficacy of radiotherapy of a human tumor xenograft, affecting tumor cells and microvessels. Strahlenther Onkol 184: 1-7. doi:10.1007/s00066-008-1001-9. PubMed: 18188516. 18188516

[24] Dagnon K, Pacary E, Commo F, Antoine M, Bernaudin M et al. (2005) Expression of erythropoietin and erythropoietin receptor in non-small cell lung carcinomas. Clin Cancer Res 11: 993-999. PubMed: 15709164. 15709164

[25] Elliott S, Busse L, Bass MB, Lu H, Sarosi I et al. (2006) Anti-Epo receptor antibodies do not predict Epo receptor expression. Blood 107: 1892-1895. doi:10.1182/blood-2005-10-4066. PubMed: 16249375. 16249375

[26] Nakamura Y, Komatsu N, Nakauchi H (1992) A truncated erythropoietin receptor that fails to prevent programmed cell death of erythroid cells. Science 257: 1138-1141. doi:10.1126/science.257.5073.1138. PubMed: 1324524. 1324524

[27] Elliott S, Busse L, McCaffery I, Rossi J, Sinclair A et al. (2010) Identification of a sensitive anti-erythropoietin receptor monoclonal antibody allows detection of low levels of EpoR in cells. J Immunol Methods 352: 126-139. doi:10.1016/j.jim.2009.10.006. PubMed: 19887071. 19887071

[28] Aapro M, Jelkmann W, Constantinescu SN, Leyland-Jones B (2012) Effects of erythropoietin receptors and erythropoiesis-stimulating agents on disease progression in cancer. Br J Cancer 106: 1249-1258. doi:10.1038/bjc.2012.42. PubMed: 22395661. 22395661 PMC3314780

[29] Vansteenkiste J, Glaspy J, Henry D, Ludwig H, Pirker R et al. (2012) Benefits and risks of using erythropoiesis-stimulating agents (ESAs) in lung cancer patients: study-level and patient-level meta-analyses. Lung Cancer 76: 478-485. doi:10.1016/j.lungcan.2011.12.015. PubMed: 22277104. 22277104

[30] Bertalanffy P, Dubsky P, Wolner E, Weigel G (1999) Alterations of endothelial nucleotide levels by mycophenolic acid result in changes of membrane glycosylation and E-selectin expression. Clin Chem Lab Med 37: 259-264. PubMed: 10353469. 10353469 10.1515/CCLM.1999.045

[31] July LV, Beraldi E, So A, Fazli L, Evans K et al. (2004) Nucleotide-based therapies targeting clusterin chemosensitize human lung adenocarcinoma cells both in vitro and in vivo. Mol Cancer Ther 3: 223-232. doi:10.4161/cbt.3.2.775. PubMed: 15026542. 15026542

[32] Higgins B, Kolinsky K, Smith M, Beck G, Rashed M et al. (2004) Antitumor activity of erlotinib (OSI-774, Tarceva) alone or in combination in human non-small cell lung cancer tumor xenograft models. Anti Cancer Drugs 15: 503-512. doi:10.1097/01.cad.0000127664.66472.60. PubMed: 15166626. 15166626

[33] Wu Y, Yang L, Hu B, Liu JY, Su JM et al. (2005) Synergistic anti-tumor effect of recombinant human endostatin adenovirus combined with gemcitabine. Anti Cancer Drugs 16: 551-557. doi:10.1097/00001813-200506000-00011. PubMed: 15846121. 15846121

[34] Döme B, Paku S, Somlai B, Tímár J (2002) Vascularization of cutaneous melanoma involves vessel co-option and has clinical significance. J Pathol 197: 355-362. doi:10.1002/path.1124. PubMed: 12115882. 12115882

[35] Döme B, Tímár J, Paku S (2003) A novel concept of glomeruloid body formation in experimental cerebral metastases. J Neuropathol Exp Neurol 62: 655-661. PubMed: 12834110. 12834110 10.1093/jnen/62.6.655

[36] Dezso K, Bugyik E, Papp V, László V, Döme B et al. (2009) Development of arterial blood supply in experimental liver metastases. Am J Pathol 175: 835-843. doi:10.2353/ajpath.2009.090095. PubMed: 19574433. 19574433 PMC2716978

[37] Bugyik E, Dezso K, Reiniger L, László V, Tóvári J et al. (2011) Lack of angiogenesis in experimental brain metastases. J Neuropathol Exp Neurol 70: 979-991. doi:10.1097/NEN.0b013e318233afd7. PubMed: 22002424. 22002424

