# Supplementary figures and images for: Erythropoietin Receptor Expression Is a Potential Prognostic Factor in Human Lung Adenocarcinoma
Source: PLoS One. 2013 Oct 14;8(10):e77459. doi: 10.1371/journal.pone.0077459 (PMC3796497; doi:10.1371/journal.pone.0077459)

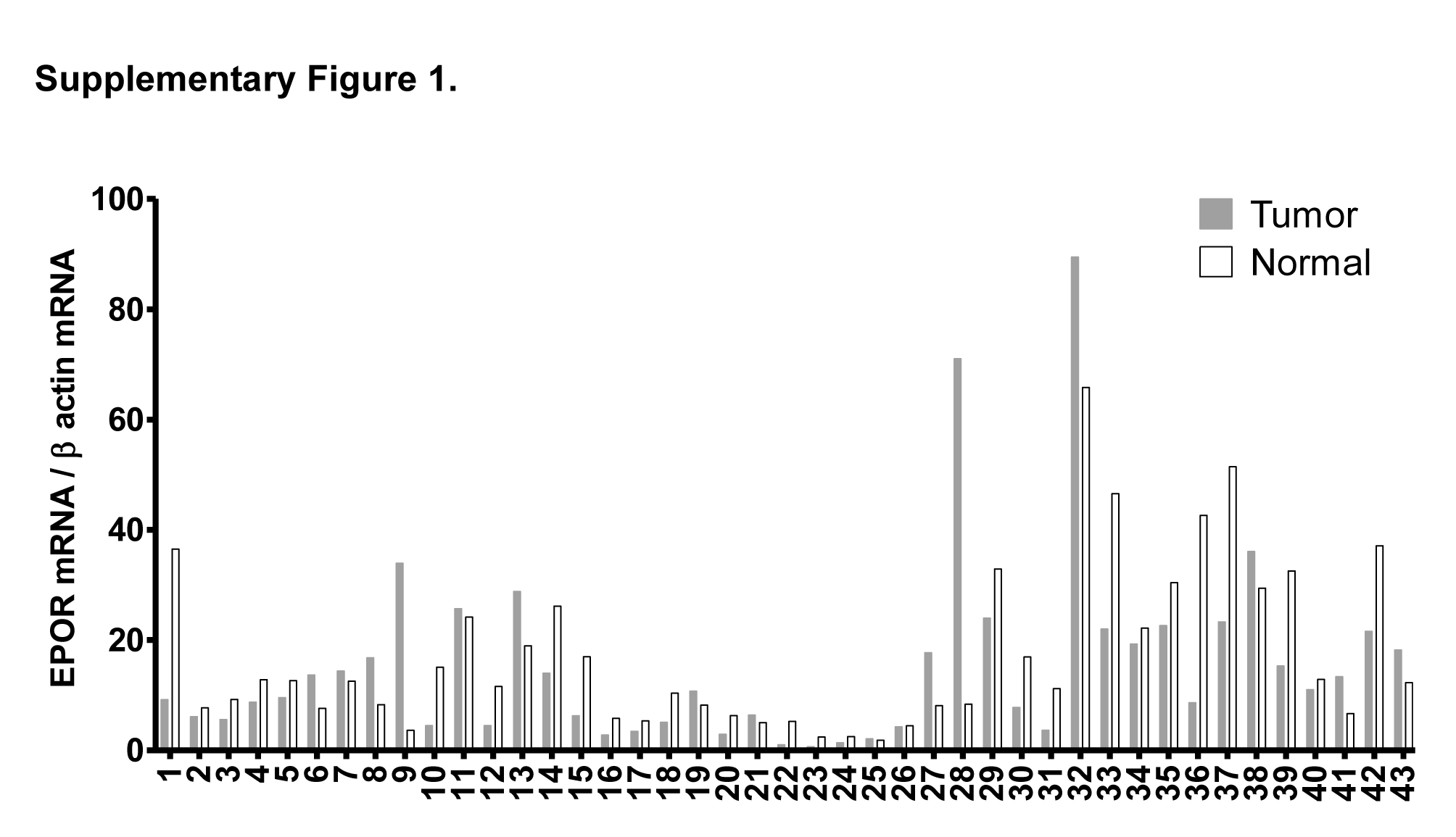

Supplement: Figure S1 — EPOR expression measured in tumoral (T) and normal (N) pairs of tissue samples of pulmonary ADC patients. For qRT-PCR measurement, RNA was isolated from bronchoscopy brushes of ADC patients. Two samples were taken in each patient, one from the tumor site (T), and an other from the tumor-free endobronchial surface (N). (TIF) [file pone.0077459.s001.tif]
